# Characteristics of the gut microbiome in women with gestational diabetes mellitus: A systematic review

**DOI:** 10.1371/journal.pone.0262618

**Published:** 2022-01-13

**Authors:** Louise Søndergaard Rold, Caspar Bundgaard-Nielsen, Julie Niemann Holm-Jacobsen, Per Glud Ovesen, Peter Leutscher, Søren Hagstrøm, Suzette Sørensen

**Affiliations:** 1 Centre for Clinical Research, North Denmark Regional Hospital, Hjørring, Denmark; 2 Steno Diabetes Centre North Denmark, Aalborg, Denmark; 3 Department of Clinical Medicine, Aalborg University, Aalborg, Denmark; 4 Department of Obstetrics and Gynecology, Aarhus University Hospital, Aarhus, Denmark; 5 Department of Clinical Medicine, Aarhus University Hospital, Aarhus, Denmark; 6 Steno Diabetes Center Aarhus, Aarhus University Hospital, Aarhus, Denmark; 7 Department of Pediatrics, Aalborg University Hospital, Aalborg, Denmark; Monash University Malaysia, MALAYSIA

## Abstract

**Background:**

The incidence of women developing gestational diabetes mellitus (GDM) is increasing, which is associated with an increased risk of type 2 diabetes mellitus (T2DM) for both mother and child. Gut microbiota dysbiosis may contribute to the pathogenesis of both GDM and the accompanying risk of T2DM. Thus, a better understanding of the microbial communities associated with GDM could offer a potential target for intervention and treatment in the future. Therefore, we performed a systematic review to investigate if the GDM women have a distinct gut microbiota composition compared to non-GDM women.

**Methods:**

We identified 21 studies in a systematic literature search of Embase and PubMed up to February 24, 2021. Data on demographics, methodology and identified microbial metrics were extracted. The quality of each study was assessed according to the Newcastle-Ottawa Scale.

**Results:**

Sixteen of the studies did find a GDM-associated gut microbiota, although no consistency could be seen. Only *Collinsella* and *Blautia* showed a tendency to be increased in GDM women, whereas the remaining genera were significantly different in opposing directions.

**Conclusion:**

Although most of the studies found an association between GDM and gut microbiota dysbiosis, no overall GDM-specific gut microbiota could be identified. All studies in the second trimester found a difference between GDM and non-GDM women, indicating that dysbiosis is present at the time of diagnosis. Nevertheless, it is still unclear when the dysbiosis develops, as no consensus could be seen between the studies investigating the gut microbiota in the first trimester of pregnancy. However, studies varied widely concerning methodology and study design, which might explain the highly heterogeneous gut microbiota compositions between studies. Therefore, future studies need to include multiple time points and consider possible confounding factors such as ethnicity, pre-pregnancy body mass index, and GDM treatment.

## Introduction

The gut microbiota plays an important role in human health, and impacts the host by influencing the immune system [1], metabolism [2], and the endocrine system [3,4]. Functionally, some bacteria have increased capacity for energy harvest, while others can induce insulin resistance [2,5,6]. Disturbance in the normal bacterial composition (dysbiosis) have been described in different diseases, and a growing body of literature supports the role of the microbiota in type 2 diabetes mellitus (T2DM) [5,7], metabolic syndrome [8], and obesity [9,10]. This is in line with fecal microbiota transplantation (FMT) studies in mice, where a dysbiotic obesity-associated microbiota can induce increased body fat storage, insulin resistance, and food energy harvest in the recipient [2,11]. This supports the theory that altered bacterial compositions can be involved in the development of metabolic disorder.

However, changes in the gut microbiota are not always associated with disease, as it also changes during pregnancy [3,12]. The mechanisms behind these microbiota alterations are not fully understood, but the dramatic hormonal changes in the pregnancy have been linked to specific bacterial changes [3]. For instance, changes in the pregnancy are characterized by an increased abundance in Actinobacteria and Proteobacteria, and a reduction in butyrate-producing bacteria and alpha diversity [12]. Proteobacteria is a gram-negative bacterium that has been linked to inflammatory-associated dysbiosis. It has been suggested that Proteobacteria induces an inflammatory response by shedding proinflammatory lipopolysaccharides [5,13,14]. Butyrate has been shown to have an anti-obesogenic effect [15], induce an anti-inflammatory response [16] and increase insulin sensitivity [17]. Overall, the gut microbiota from women in the first trimester is similar to non-pregnant controls, whereas the third-trimester gut microbiota resembles that of persons with obesity or metabolic syndrome [3,12] Animal studies have further revealed, that transplantation of gut microbiota from women in the third trimester of pregnancy induced low-grade inflammation, adiposity, insulin resistance, and hyperglycemia in mice, resembling changes seen in metabolic syndrome [12]. However, unlike in metabolic syndrome, the metabolic changes are necessary in a healthy pregnancy. At the beginning of the pregnancy, insulin sensitivity increases resulting in increased glucose storage to meet the energy demands later in pregnancy [18,19]. However, as the pregnancy progresses, a reduction in insulin sensitivity is seen [18]. This, together with an increase in endogenous glucose production, leads to elevated blood glucose levels, which ensures the demands of the growing fetus [20].

Pregnancy, therefore, induces a metabolic shift, which is beneficial for the growing fetus. However, in some cases an abnormally increased insulin resistance [18] and/or dysfunction in insulin secretion are seen, introducing a hyperglycemic state termed gestational diabetes mellitus (GDM) [21–23]. GDM is defined as glucose intolerance with onset during pregnancy and is associated with an increased risk of obstetric and neonatal complications [24–26]. The GDM women are at higher risk of hypertension, pre-eclampsia, and the need for cesarean section. For the child, there is an increased risk of preterm delivery, being large for gestational age, shoulder dystocia, and neonatal hypoglycemia [25–27]. Furthermore, GDM has also been associated with long-term effects, as both mother and child are at increased risk of developing T2DM [28–36]. Early intervention against GDM is, therefore, important to reduce the risk of complications in mother and child. Several risk factors have been identified for the development of GDM; for instance, obesity, family history of T2DM, previous history of GDM, advanced age, diagnosis with polycystic ovary syndrome, and previous macrosomia [37–39]. However, GDM does not usually cause any noticeable symptoms, and the diagnosis is, therefore, based on screening pregnant women with an oral glucose tolerance test (OGTT).

As the gut microbiota may contribute to the metabolic changes during pregnancy, it is possible that a dysbiotic gut microbiota contributes to the unwanted metabolic changes seen in GDM. Furthermore, a FMT study in mice has shown that it is possible to induce metabolic changes in the recipient when transferring bacteria from GDM women, which suggest that the gut microbiota influences the metabolic changes in GDM [40]. Nevertheless, there are uncertainties regarding when the dysbiosis develops, and whether the altered microbiota is part of the cause or the consequence of the GDM development.

Therefore, this review aims to provide an overview and comparison of previous studies investigating the association between gut microbiota and GDM.

## Methods

### Search protocol

The systematic literature search was conducted according to the Preferred Reporting Items for Systematic Reviews and Meta-analyses (PRISMA) guidelines [41]. The search was performed on February 24^th^, 2021 in Pubmed and Embase, with no restriction on publication year. Search strings for both databases are shown in S1 File.

### Eligibility criteria

Eligible studies were original studies on women who, at some point, were diagnosed with GDM and where an assessment of the gut microbiota composition was performed. GDM could be diagnosed based on national, international or study specific criteria. Only articles written in Danish or English were included. It was further required that the studies included a control group and that a minimum of 10 GDM women were assessed. Case reports, animal studies, reviews, and conference abstracts were excluded. Intervention studies were likewise excluded apart from data from baseline samples taken before intervention.

### Study selection

Articles were screened, and duplicates removed, using the systematic reviews web app Rayyan (http://rayyan.qcri.org). The articles were initially screened by title and abstract, according to eligibility criteria, by two independent researchers (SES and LR). Next, the included articles were subjected to whole-paper examination. Disagreement was resolved through discussion between SES and LR.

### Data extraction

The following data was extracted from included articles: sample size, age, ethnicity (country of study site), diagnosis criteria, pre-pregnancy BMI, fasting glucose levels, sample storage, DNA extraction method, primer choice, sequencing platform, bioinformatics platform, alpha- and beta diversity measures, and bacteria with significant differential relative abundance in cases vs. controls. The level of significance was based on the p-values given in the individual studies. Reported correlations between specific bacteria and host parameters (maternal blood analyses and weight gain during pregnancy) were also extracted from the included articles.

### Meta analyses on age, pre-pregnancy BMI and fasting glucose levels

To investigate whether studies from different regions had differences in participants included, we performed a meta-analysis on participant number, age, pre-pregnancy BMI, and fasting glucose levels. All statistics were performed in R version 4.0.3 (https://www.r-project.org/) through Rstudio IDE (http://www.rstudio.com/). Distribution and variance were tested using Shapiro-Wilks test and Bartlett’s test, respectively. If normal distributed, we used Student’s t test or one-way ANOVA with Tukey’s post hoc test. If nonparametric, we used Mann-Whitney U test or Kruskal-Wallis test with Dunn’s post hoc test and Benjamini-Hochberg’s procedure to adjust for false-discovery rate. For both p-values and false-discovery rates, a cutoff of <0.05 was considered significant.

### Quality assessment

The quality of the included studies was assessed using the Newcastle-Ottawa Scale (NOS) for case-control studies [42]. NOS is based on three criteria: 1) selection, 2) comparability and 3) exposure. The selection criteria included 1) adequate definition of the cases, 2) representativeness of the cases, 3) selection of controls, 4) definition of controls. The comparability criterium was based on the comparability of case and controls according to the study design and analysis. The exposure criterium was based on 1) ascertainment of exposure, 2) same method of ascertainment for cases and controls, 3) non-response rate.

A quality score ranging from 0 to 10 was obtained by the use of a rating algorithm previously described: 0–5 (poor), 6–7 (moderate), and 8–10 (high) [43,44]. The quality score is given based on how well the study describes and investigates differences between GDM and non-GDM participants. Therefore, a study which primary focus is not on GDM, can be well performed, but still receive a low score in this systematic review.

## Results

### Study selection

**Fig 1:**
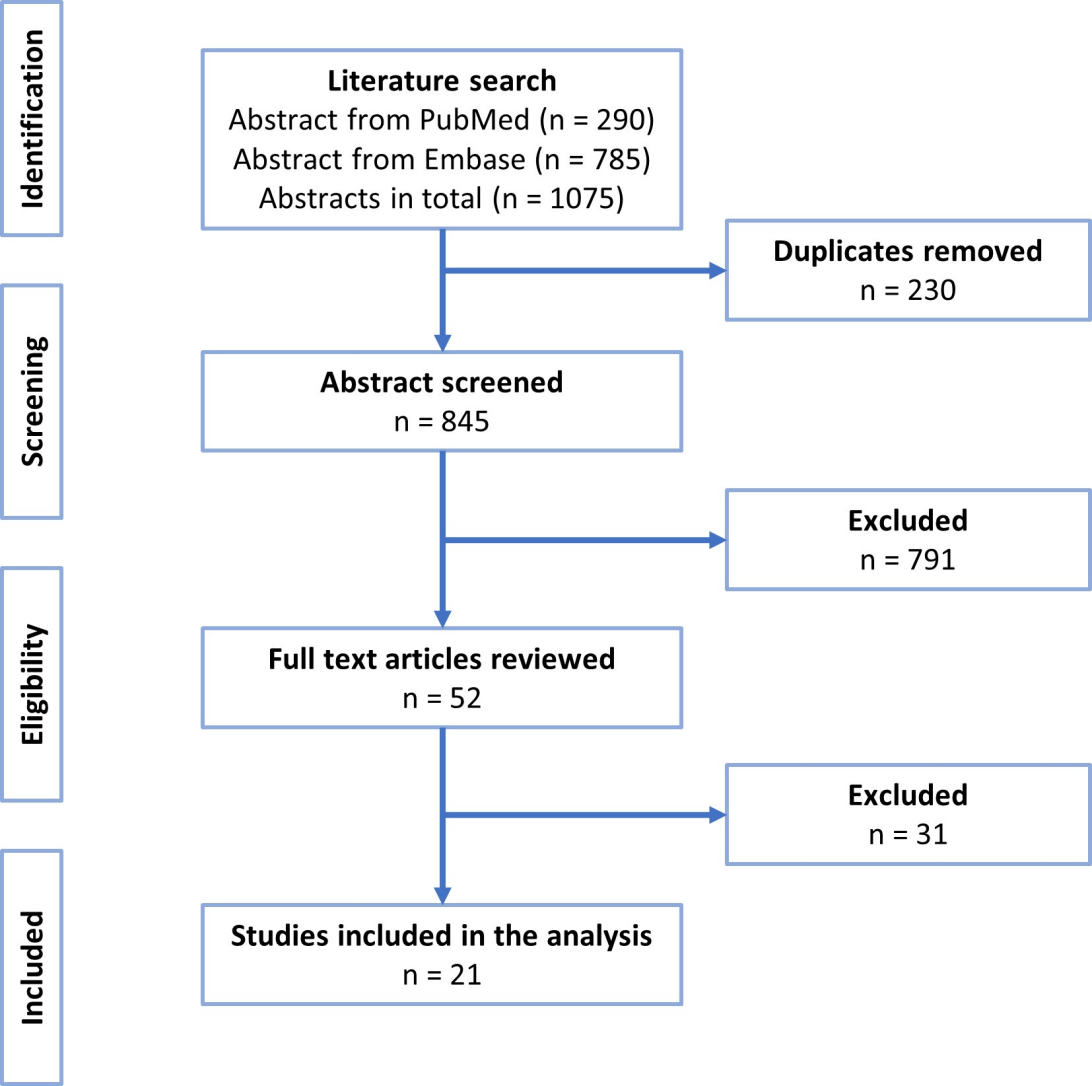
PRISMA flow diagram of study selection.

The literature search resulted in the identification of 290 articles from PubMed and 785 articles from Embase. After the removal of duplicates, the total number of articles was reduced to 845. These were screened based on title and abstract and resulted in 52 articles for full-text screening. Twenty-one articles remained after the full-text screening and were included in this systematic review (Fig 1).

### Study characteristics

**Fig 2:**
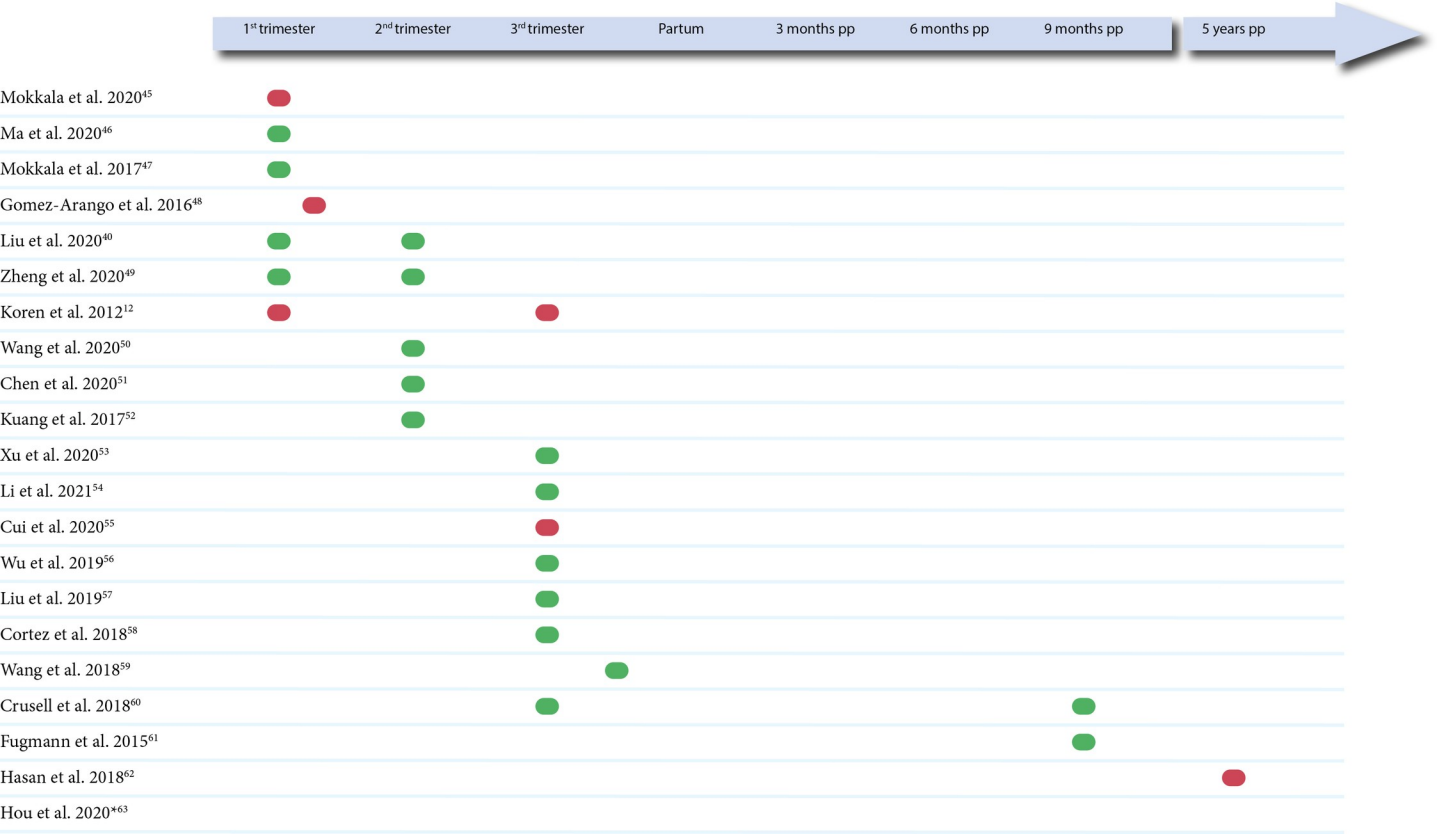
Time-point for microbiota analyses. Green symbol indicates that the study found a statistically significant difference between GDM and non-GDM women in either alpha diversity, beta diversity, or relative bacterial abundance. Red symbol indicates that the study did not find a statistically significant difference between the GDM and non-GDM women. The level of significance for each study can be seen in Table 2. *No information regarding the time-point for microbiota analysis.

Among the included studies, 16 were cross-sectional studies investigating the gut microbiota at a specific time-point (Fig 2). Among the remaining five studies, one of the studies [45] was an interventional study from which we only included the baseline data in the analysis and four studies were longitudinal studies with two or three collection time points. Almost all the studies investigated the gut microbiota during pregnancy (seven studies in the first trimester [12,40,45–49], five studies in the second trimester [40,49–52] and nine studies in the third trimester [12,53–60]) with only three studies investigating the gut microbiota postpartum [60–62]. All microbiota analyses in the first trimester were made with a prognostic purpose, as none of the included women were diagnosed with GDM in the first trimester (Table 1). In most of the studies investigating the second trimester, sample collection and the diagnosis time point were overlapping. An exception was in Chen *et al*. 2020 [51] where the samples were collected in gestational week 22–24 and the diagnosis time was in gestational week 25–26.

**Table 1:** Demographic and clinical characteristics of the included studies.

Study	Nationality	Comparability	Group	Sample size (n)	Age (years)	Pre-pregnancy BMI	Fasting glucose (mmol/l)	Diagnostic criteria (Time of diagnosis)	Antibiotics	GDM treatment
Mokkala et al.2020 [45]	Finland	Included overweight/obese women. Excluded women with early GDM. Adjusted for pre-pregnancy BMI and previous GDM.	GDM:	67	eGDM: 31.7±6.2	eGDM 32.5±5.5	NA	Finnish Current Care guidelines and IADPSG (GW 14±1,9 or GW26,3±2,0)	No use within 8 weeks before sample collection	Excluded women taking insulin or metformin
					mGDM: 31.3±4.4	mGDM 30.1±4.8	NA			
			Non-GDM:	203	30.7±4.3	28.7±3.5	NA			
Ma et al.2020 [46]	China	Matched for age, gestational age, and sample collection.	GDM:	70	31.0 (28.8–34.0)*	NA	4.82 (4.54–5.15)*	IADPSG (GW 24–28)	No use in the pregnancy	NA
			Non-GDM:	70	32.5 (29.0–35.0)*	NA	4.62(4.3–4.74)*			
Mokkala et al.2017 [47]	Finland	Included obese women. Adjusted for pre-pregnancy BMI and intervention.	GDM:	15	29.3±3.2	39.8±3.5	5.0±0.4	Finnish Current Care guidelines (GW 25,5±2,2)	Not an exclusion criterion	NA
			Non-GDM:	60	30.3±4.6	30±4.5	4.7±0.3			
Gomez-Arango et al.2016 [48]	Australia	Included overweight/obese women. Excluded women with early GDM. Matched for age, BMI and ethnicity.	GDM:	26	NA	OW = 8 O = 18	NA	IADPSG (GW28)	NA	Excluded women taking agents affecting the glucose metabolism
			Non-GDM:	44	NA	OW = 21 O = 23	NA			
Liu et al.2020 [40]	China	Matched for age and pre-pregnancy BMI. Samples are collected before treatment.	GDM:	45	32.8±3.3	NA	5.1±0.6	IADPSG (GW 24–28)	NA	Samples are collected before treatment
			Non-GDM:	45	32.8±3.9	NA	4.7±0.4			
Zheng et al.2020 [49]	China	Control for BMI, total cholesterol, and total triglyceride.	GDM:	31	32.58±4.1	22.57±5.7	4.84 (4.53–5.20)*	IADPSG (GW 24–28)	No use within the last 2 months	Excluded women taking drugs affecting the gut microbiota within 2 months prior to entry
			Non-GDM:	103	31.79 ±3.7	21.32±3	4.46(4.26–4.66)*			
Koren et al.2012 [12]	Finland	NA	GDM:	15	NA	NW = 7, OW = 6, O = 2	NA	GCT in women at risk: 0-h ≥4.8 mmol/l combined with 1-h value ≥10.0 mmol/L and/or 2-h value ≥8,7 mmol/L (GW 26–28)	Not an exclusion criterion	NA
			Non-GDM:	76	NA	NW = 46, OW = 24, O = 6	NA			
Wang et al.2020 [50]	China	Adjusted for BMI and age.	GDM:	59	30.56±4.24	NA	4.91±0.39	IADPSG (GW 24–28)	No use 1 month before sample collection	NA
			Non-GDM:	48	29.19±3.04	NA	4.70±0.52			
Chen et al.2020 [51]	China	Matched for pre-pregnancy BMI, parity, and age.	GDM:	110	<30 = 48, 30–35 = 43, ≥35 = 19	21.4±3.2	4.4±0.4	IADPSG (GW 25–26)	No use within the last 3 months	NA
			Non-GDM:	220	<30 = 97, 30–35 = 85, ≥35 = 38	21.4±3.1	4.2±0.3			
Kuang et al.2017 [52]	China	NA	GDM:	43	30.5±3.3	21.9±3.1	4.7±0.5	IADPSG (GW 21–29)	No use 1 month before sample collection	None of the women were treated with insulin or glyburide
			Non-GDM:	81	28.8±3.1	20.2± 2	4.3±0.3			
Li et al.2021 [54]	China	NA	GDM:	23	29.80±2.19	23.64±1.36	5.29±0.58	IADPSG (NA)	No use within the last 4 weeks	NA
			Non-GDM:	29	29.00±1.88	21.39± 1.37	4.44±0.42			
Xu et al.2020 [53]	China	Adjusted for obesity and insulin usage.	GDM:	30	33.7± 4.7	24±3.6	NA	IADPSG (GW 24–28)	No use within the last 2 weeks	Insulin usage was recorded
			Non-GDM:	31	32.3±4.3	22±3.1	NA			
Cui et al.2020 [55]	China	NA	GDM:	21	NA	NA	4.4±0.98	Fasting glucose ≥5.1 mmol/L and/or HbA1c ≥6% (T3)	No use within the last month	NA
			Non-GDM:	36	NA	NA	NA			
Wu et al.2019 [56]	China	NA	GDM:	23	36 (32–38.5)◊	22,58 (19.42–25.58)◊	4,8(4.5–5.1)◊	IADPSG (GW 24–28)	No use within the last 6 months	Diet and exercise counseling, and insulin if needed
			Non-GDM:	26	32.5 (30–35)◊	20,96 (19.70–22.17)◊	4,185(4.1–4.3)◊			
Liu et al.2019 [57]	China	Included only women with GDM risk factors.	GDM:	11	29.3±0.9•	NA	5.0±0.09•	IADPSG (GW 27–33)	No use within the last 3 months	NA
			Non-GDM:	11	28.2±0.08•	NA	4.5±0.08•			
Cortez et al.2018 [58]	Brazil	NA	GDM:	26	35.07±3.75	NW = 4 OW = 8 O = 11	NA	IADPSG (NA)	NA	Diet counseling and insulin if necessary
			Non-GDM:	42	28.23±5.68	NW = 19 OW = 14 O = 9	NA			
Wang et al.2018 [59]	China	NA	GDM:	74	NA	NA	NA	IADPSG (24–28)	NA	NA
			Non-GDM:	73	NA	NA	NA			
Crusell et al.2018 [60]	Denmark	Included only women with GDM risk factors. Adjusted for pre-pregnancy BMI.	GDM:	50 (43)	34.4±4.4	29.3±5.6	5.2±0.4	IADPSG (GW 27–33)	No use within the last 2 months	None of the women were treated with anti-diabetic drugs
			Non-GDM:	161 (82)	33.3±4.6	27.1±4.8	4,6±0.2			
Fugmann et al. 2015 [61]	Germany	NA	GDM:	42	37 (34–39)*	NA	NA	IADPSG (GW 23-)	No use 14 days before sample collection	NA
			Non-GDM:	35	36 (32–38)*	NA	NA			
Hasan et al.2018 [62]	Finland	Included only high-risk women.	GDM:	60	39.2±4,4	≥30	5.7(0,5)	Finnish Current Care guidelines (Enrollment or GW 24–28)	NA	Excluded women taking agents affecting the glucose metabolism
			Non-GDM:	68	37.7±5.3	≥30	4.9(0,4)			
Hou et al. 2020 [63]	China	NA	GDM:	61	NMA 28.27±2.37	NMA 24.12±4.23	NA	IADPSG (GW 24–28)	No use within the last month	Excluded women taking agents affecting the glucose metabolism
					AMA 36.23±3.03	AMA 23.83±3.48	NA			
			Non-GDM:	50	30.23±3.03	22.53±2.99	NA			

eGDM, early onset GDM; mGDM, mid-pregnancy onset GDM; NMA, normal maternal age; AMA, advanced maternal age; NW, normal weight; OW, overweight; O, obese; IADPSG, International Association of Diabetes in Pregnancy Study Group; GCT, glucose challenge test; GW, gestational week; NA, not available; *, median ± IQR; •, mean ± SEM; ◊, mean ± IQR. If no symbol is applied the value is indicated as mean ± SD. IADPSG: One or more of the values from a 75-g OGTT must be equaled or exceeded 5.1 mmol/l (FBG), 10.0 mmol/l (1-h) or 8.5 (2-h) to diagnose GDM. Finnish Current Care guidelines: One or more of the values from a 75-g OGTT must be equaled or exceeded 5.3 mmol/l (FBG), 10.0 mmol/l (1-h) or 8.6 (2-h) to diagnose GDM.

**Fig 3:**
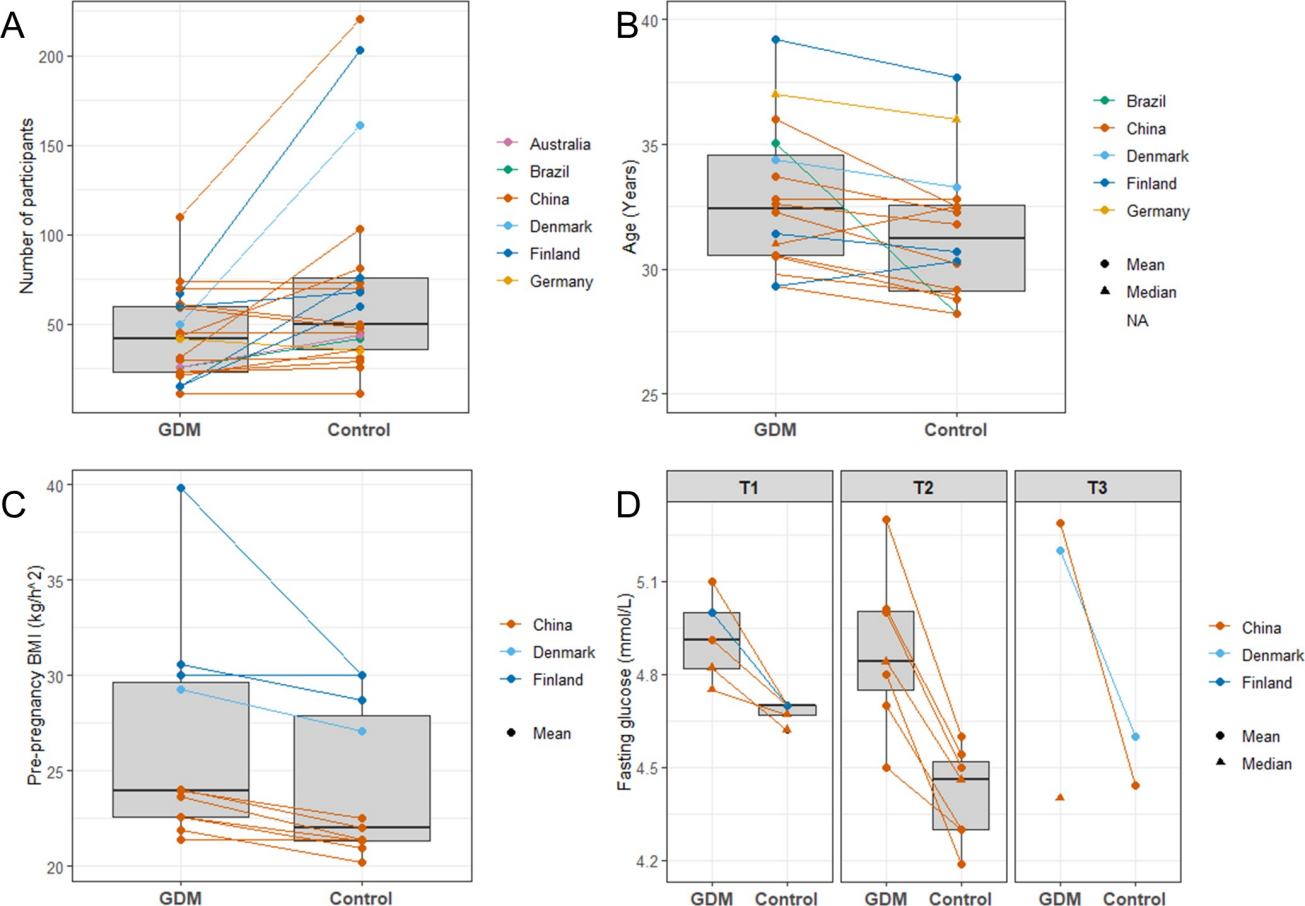
Box plots comparing GDM and non-GDM women in the included studies. (A) Number of participants, (B) Age, (C) Pre-pregnancy BMI, and (D) Fasting glucose measured in the different trimesters of pregnancy. T1, first trimester; T2, second trimester; T3, third trimester.

Demographics and clinical characteristics of participants are shown in Table 1. The total number of participants with GDM was 945 (range = 11 to 110), whereas the total number of non-GDM pregnant control participants was 1594 (range = 11 to 220). Studies from China were highly represented, as 13 of the 21 studies were conducted in China [40,46,49–57,59,63], and the remaining were performed in Finland [12,45,47,62], Brazil [58], Denmark [60], Australia [48], and Germany [61]. Selection criteria for included women differed, as five of the studies only included women at risk of developing GDM, whereas the remaining studies included women from all risk groups (Table 1). In most studies, the GDM women were older than the controls (Table 1 and Fig 3B, respectively), but only three studies reported a significant difference in age between the groups [52,56,58]. Five studies found a significantly higher pre-pregnancy BMI in the GDM women [52,54,58,60,63]. However, only 10 of the studies either matched their groups or adjusted for pre-pregnancy BMI and age when performing their analyses (Table 1). Furthermore, the Chinese women also had a lower pre-pregnancy BMI (Table 1 and Fig 3C, respectively) compared to the Finnish (p = 0.0003) and Danish women (p = 0.062). Most of the studies used the International Association of Diabetes in Pregnancy Study Group’s (IADPSG) diagnosis criteria [38] to diagnose GDM women, but the Finnish studies and one of the Chinese studies used other criteria [12,45,47,55,62]. Another difference in the study designs was the history of antibiotic use, as some studies excluded women that had used antibiotics within six months before sample collection, whereas others did not exclude women based on the use of antibiotics (Table 1). The GDM treatment regimens also differed between the studies, as some of the studies included women receiving both lifestyle counseling and antidiabetic drugs [53,56,58], while others excluded women taking antidiabetic treatment such as insulin and metformin [45,48,52,60,62,63].

### Storage and sample handling

The included studies were highly different regarding sample handling and methodology for microbiota analyses (Table 2). Following sample collection, most of the studies stored the samples at either -20˚C or -80˚C, while three studies used storage buffers [49,61,62], two studies stored the samples at 4˚C [48,57], and one study handled the samples immediately [55]. DNA extraction was performed with a wide variety of extraction kits, and only the QIAamp DNA Stool Mini kit was used in more than two studies [52,58,59]. 16S rRNA gene sequencing was applied to assess the gut microbiota in 18 studies, while three studies used metagenomic sequencing [45,52,56]. Different 16S rRNA variable regions were targeted in DNA sequencing, but the V3-V4 region was the most applied [40,49–51,53,54,57,59]. A variety of different databases were used to assign taxonomy, but the most used were SILVA [40,45,46,57,58,62] and Greengenes [12,48,51,54,55,59]. Most of the studies that applied 16S rRNA sequencing used a threshold of 97% to cluster their 16S rRNA sequences [12,40,48–50,54,55,57–62], though a few studies used 99 or 100% sequence similarity for clustering [46,51]. Furthermore, three studies utilized whole genome sequencing [45,52,56]. All the metagenomic studies as well as three studies utilizing 16S rRNA gene sequencing, assigned taxa to species level. Conversely, all studies but one [63], included taxonomic assignment at genus level. The threshold for level of significance was also different between the included studies. Thirteen studies described correcting for multiple comparisons [12,45,47–49,51–53,55,59–62], mostly through the use of Benjamini-Hochbergs procedure to adjust for FDR [45,47,48,52,55,59–62]. Different cutoff values were used, including 0.05 [12,40,46,48,50–55,57,62,63], 0.1 [47,49,59,60], or 0.2 [45]. Several studies further utilized LEfSe analysis, with a LDA>2.0 considered significant [40,46,50].

**Table 2:** Handling of samples from GDM and non-GDM participants in the included studies.

Study	Sample storage	DNA extraction	Sequencing technique	Target	Reference database	Clustering	Taxonomic assignment	Diversity	Threshold for significant differences
Mokkala et al. 2020 [45]	-20˚C	Bead beating and GTX stool extraction kit	Illumina HiSeq	Metagenomics	Silva	NA	P, C, O, F, G, S	α: Observed species, Shannon, β: Bray-Curtis	Benjamini-Hochberg FDR adjusted p-value<0.2
Ma et al.2020 [46]	Frozen at -18˚C, transported on dry ice, stored at -80˚C	QIAamp fast dna stool mini kit	Illumina HiSeq 2500	16S rRNA V4	Silva	100% OTU	P, F, G	α: Simpson, Chao1, Shannon, Heip e, Ace, Dominance. β: Jaccard, Bray-Curtis, unweighted and weighted UniFrac distances	P<0,05 LDA>2.0 for LEfSe
Mokkala et al. 2017 [47]	NA	NA	NA	NA	NA	OTU (% not specified)	P, C, O, F, G, S	α: NA β:NA	Benjamini-Hochberg FDR adjusted p-value<0.1
Gomez-Arango et al.2016 [48]	Refrigerated, stored at -80˚C (within 1 day)	AllPrep DNA extraction kit	Illumina MiSeq	16S rRNA V6-V8	Greengenes	97% OTU	P, C, F, G	α: NA β: Weighted UniFrac distances	Benjamini-Hochberg FDR adjusted p-value<0,05
Liu et al.2020 [40]	Stored at -80˚C (within 3 hours)	PowerFecal DNA Kit	Illumina HiSeq 2500	16S rRNA V3-V4	Silva	97% OTU	P, G	α: Chao1, Shannon β: Bray-Curtis	P<0,05 LDA>2.0 for LEfSe
Zheng et al. 2020 [49]	PSP Spin stool DNA Plus kit, transported on dry ice (immediately), stored at -80˚C	TIANamp stool DNA kit	Illumina MiSeq	16S rRNA V3-V4	RDP	97% OTU	P, C, O, F, G	α: Shannon β: Weighted UniFrac	FDR adjusted p-value <0.1
Koren et al. 2012 [12]	Frozen at -18˚C, transported on dry ice, stored at -80˚C	PowerSoil-htp DNA isolation kit and bead-beating	Roche 454 FLX and Titanium chemistry	16S rRNA V1-V2	Greengenes	97% OTU	P, O, F, G	α: Phylogenetic diversity, Pilou indices β: Unweighted and weighted UniFrac	FDR adjusted p-value<0,05
Wang et al. 2020 [50]	Stored at -80˚C	OMEGA-soil DNA kit	Illumina MiSeq	16S rRNA V3-V4	NA	97% OTU	P, F, G	α: Phylogenetic diversity, Chao1, Shannon, Ace β: Euclidean	P<0,05, LDA>2.0 for LEfSe
Chen et al. 2020 [51]	Frozen at -20˚C, stored at -80˚C	QIAamp Fast DNA Stool Mini Kit	Illumina MiSeq	16S rRNA V3-V4	Greengenes	99% OTU	P, C, O, F, G	α: Shannon β: Weighted UniFrac	FDR adjusted p-value <0,05 LDA>2.0
Kuang et al. 2017 [52]	Frozen at -20˚C, stored at -80˚C (within 24 hours)	QIAamp DNA stool mini kit	Illumina HiSeq	Metagenomics	NCBI genome database	NA	P, C, O, F, G, S	α: Observed species, Shannon β: Bray-Curtis	Benjamini-Hochberg adjusted p-value < 0,05
Li et al. 2021 [54]	Transported in sampling box, stored at -80˚C. DNA extraction within 48 hours	BGI Stool Genome Extraction Kit	Illumina Hiseq 2500 PE250	16S rRNA V3-V4	Greengenes	97% OTU	P, C, O, F, G, S	α: Observed species, Simpson, Chao1, Shannon, Ace β: Euclidean	P<0,05 LDA: NA
Xu et al. 2020 [53]	Frozen at -18˚C, transported on dry ice (within 2 hours), stored at -80˚C.	Bead beating method	Illumina Hiseq 2500 PE250	16S rRNA V3-V4	NA	OTU (% not specified)	C, O, F, G	α: Simpson, Chao1, Shannon, Ace, β: Bray-Curtis, weighted and unweighted UniFrac	FDR adjusted p-value <0,05
Cui et al. 2020 [55]	Fresh stool sample	Custom DNA extraction protocol	Illumina MiSeq	16S rRNA V4	Greengenes	97% OTU	P, G	α: NA β: Bray-Curtis	Benjamini-Hochberg adjusted p-value <0,05
Wu et al. 2019 [56]	Stored at -80˚C (immediately)	MoBio powerfecal DNA kit	Illumina HiSeq 2500	Metagenomics	HMP	NA	P, G, S	α: Shannon β: Bray-Curtis	LDA>2.0 Cutoff or method for adjusted p-value is not defined.
Liu et al. 2019 [57]	Refrigerated, stored at -80˚C (within 1 day)	PowerMax (stool/soil) DNA isolation kit	Illumina HiSeq4000	16S rRNA V3-V4	Silva	97% OTU	P, C, O, F, G, S	α: Observed species, Phylogenetic diversity, Chao1 β: UniFrac distance metrics	P<0,05 No information on method to handle multiple comparisons
Cortez et al. 2018 [58]	Frozen at -20˚, stored at -80˚C.	QIAamp DNA stool mini kit	Illumina MiSeq	16S rRNA V4	Silva	97% OTU	P, G	α: Simpson, Chao1, Shannon β: Weighted and unweighted UniFrac	P-values < 0.01
Wang et al. 2018 [59]	Frozen at -20˚, stored at -80˚C.	QIAamp DNA Stool Mini Kit	Illumina HiSeq 2500	16S rRNA V3-V4	Greengenes	97% OTU	P, C, O, F, G	α: NA β: Unweighted UniFrac	Benjamini-Hochberg FDR adjusted p-value<0.1, LDA>2.0 for PLS-DA
Crusell et al.2018 [60]	Frozen at -18˚C, transported on dry ice, stored at -80˚C (within 48 hours)	NucleoSpin Soil kit	Illumina MiSeq	16S rRNA V1-V2	RDP classifier	97% OTU	P, C, O, F, G	α: Observed species, Shannon, Pilou indices β: Weighted UniFrac	Benjamini-Hochberg FDR adjusted p-value<0.1
Fugmann et al. 2015 [61]	Stool DNA stabilizer, mailed (within 1 day), stored at -80˚C	PSP® Spin Stool DNA Plus Kits	Illumina MiSeq	16S rRNA V4	The RDP classifier eztaxon	97% OTU	P, C, O, F, G, S	α: Simpson, Chao1, Shannon β: Bray-Curtis	Benjamini-Hochberg FDR adjusted p-value. Cutoff for adjusted p-valued is not specified.
Hasan et al. 2018 [62]	Stool DNA stabilizer, domestic freezer, stored at -80˚C	PSP® Spin Stool DNA Plus Kits	Illumina MiSeq	16S rRNA V1-V3	Silva	97% OTU	P, C, O, F, G	α: Inverse Simpson and Shannon indices, Observed species β: Bray-Curtis	Benjamini-Hochberg FDR adjusted p-value<0,05
Hou et al. 2020 [63]	Stored at -80˚C as soon as possible	Stool DNA extraction kit	Illumina HiSeq 200	16S rRNA V4	NA	OTU (% not specified)	P	α: NA β:NA	P<0,05

NA, not available; OTU, Operational Taxonomic Unit; α, alpha diversity; β, beta diversity; P, Phylum; C, Class; O, Order; F, Family; G, Genus; S, Species; FDR, False Discovery Rate.; LDA, Linear Discriminant Analysis; LEfSe, Linear discriminant analysis Effect Size.

### Studies investigating the gut microbiota in GDM women showed very inconsistent results

Even though 16 of the studies did find an association between GDM and composition of gut microbiota, the specific results varied between the studies (Fig 2 and Table 3, respectively). Shannon diversity was the most used analysis to investigate alpha diversity either alone or in combination with other analyses such as Chao1 richness, observed species, Simpson, and ACE (Table 2). Six of the 21 studies found a significantly reduced alpha diversity in the gut microbiota of GDM women compared to controls [40,46,50–52,57]. Most of the studies used Bray-Curtis to investigate the beta diversity [40,45,46,52,53,55,56,61,62] but many of the studies also used unweighted [12,46,53,58,59] and/or weighted UniFrac [12,46,48,49,51,53,58,60]. For beta diversity, nine studies reported that the gut microbiota varied between GDM and non-GDM women [40,46,50–54,56,59], whereas the remaining studies did not observe any differences. Furthermore, the included studies differed in the taxonomic level they used when analyzing significant differences in relative abundances between the cases and controls. All of the included studies identified bacteria at genus-level, except Hou *et al*. 2020 [63] that performed their analysis at phylum level. Furthermore, 22 bacteria were reported to be statistically significant different in relative abundance between cases and controls in three or more of the included studies (see Table 3). Three studies found an increased relative abundance of the order Coriobacteriales in the GDM group. Furthermore, the relative abundance of 11 genera were different between GDM and non-GDM women in three or more microbiota comparisons. Only *Collinsella* and *Blautia* showed a tendency (only seen in four microbiota comparisons) to be increased in GDM women, whereas the remaining genera were significantly different in opposing directions.

**Table 3:** Alterations of the gut microbiota in the GDM women compared to the non-GDM women.

Studies	Liu et al. 2020 [40]	Mokkala et al. 2020 [45]	Zheng et al. 2020 [49]	Ma et al. 2020 [46]	Mokkala et al. 2017 [47]	Gomez-Arango et al. 2016 [48]	Koren et al. 2012 [12]	Wang et al. 2020 [50]	Liu et al. 2020 [40]	Chen et al. 2020 [51]	Zheng et al. 2020 [49]	Kuang et al. 2017 [52]	Li et al. 2021 [54]	Xu et al. 2020 [53]	Cui et al. 2020 [55]	Wu et al. 2019 [56]	Liu et al. 2019 [57]	Cortez et al. 2018 [58]	Wang et al. 2018 [59]	Crusell et al. 2018 [60]	Koren et al. 2012 [12]	Crusell et al. 2018 [60]	Fugmann et al. 2015 [61]	Hasan et al. 2018 [62]	Hou et al. 2020 [63]		
**Time-point**	First trimester/ early pregnancy							Second trimester						Third trimester								Postpartum			?	Total	
**Beta diversity**																										Total D	Total N
Beta diversity	N	N	N	D		N	N	D	D	D	N	D	D	D	N	D		N	D	N	N	N	N	N		9	13
**Alpha diversity**																										Total ↓	Total ↑
Richness	↓	N		↓				N	↓	↓		↓	N	N			↓	↑		N		N	N	N		6	1
Evenness		N	N	↓↑			N	↓	↓	↓	N	↓	↑	N		N	↓	N		N		N	N	N		6	2
**Phylum level**																										Total ↓	Total ↑
*Actinobacteria*										↓										↑		↑			↑	1	3
*Bacteroidetes*									↓	↑			↓												↑	2	2
*Firmicutes*										↓			↑										↓		↓	3	1
**Class level**																										Total ↓	Total ↑
*Actinobacteria*										↓										↑		↑				1	2
*Bacteroidia*					↓					↑			↓													2	1
*Clostridia*					↑					↓			↑													1	2
**Order level**																										Total ↓	Total ↑
*Coriobacteriales*													↑							↑		↑				0	3
*Clostridiales*					↑							↓	↑													1	2
**Family level**																										Total ↓	Total ↑
*Coriobacteriaceae*												↓	↑							↑		↑				1	3
*Lachnospiraceae*								↓↑					↑					↑								1	3
*Ruminococcaceae*					↑			↓					↑					↑								1	3
**Genus level**																										Total ↓	Total ↑
*Collinsella*													↑					↑		↑		↑				0	4
*Eggerthella*											↑	↓										↓				2	1
*Bacteroides*										↑								↑		↓		↓				2	2
*Ruminococcus2*								↓												↑		↓				2	1
*Blautia*									↑				↑					↑		↑						0	4
*Coprococcus*			↓								↓		↑													2	1
*Roseburia*								↑				↓						↑								1	2
*Faecalibacterium*								↓	↑								↓			↓↑		↓↑				4	4
*Fusobacterium*												↓							↑			↓				2	1
Sutterella										↑			↓							↓						2	1
*Akkermansia*								↑	↓								↑									1	2

Bacteria are illustrated if they are significantly different in relative abundance between cases and controls in 3 or more of the included studies. ↑ indicates a higher alpha diversity or bacteria are more abundant in the GDM group compared to the control group. ↓ indicates a lower alpha diversity or bacteria are less abundant in the GDM group compared to the control group. ↓↑ indicates significantly differences in opposing directions in alpha diversity or specific bacteria abundance. D indicates a significant difference in beta diversity between the GDM and the control group. N indicates no difference in beta diversity between the groups. A question mark (?) indicates unknown sample collection time point.

Some differences could be seen, when comparing the trimesters separately (see Table 3). The first trimester/early pregnancy gut microbiota was investigated by seven of the included studies, where four of these found an altered gut microbiota in women before GDM development [40,46,47,49]. However, no consistency could be seen in neither diversity measurements nor difference in the relative abundance of specific bacteria between the groups. The second trimester of pregnancy showed the highest agreement between the studies, as five studies, all Chinese, investigated the gut microbiota in the second trimester, and all reported a significant difference between GDM and non-GDM women [40,49–52]. Apart from the study by Zheng et *al*. [49], all studies showed a reduced alpha diversity in GDM women and a difference in beta diversity between the groups (Table 3). Furthermore, most of the differences in individual bacteria, reported in more than three studies, were observed in the second trimester (Table 3). Even though more of the studies agreed that the relative abundance of a bacterium differed between the groups, there was no agreement, whether it was increased or reduced in GDM women. In the third trimester of pregnancy, seven out of nine studies found a difference between the GDM women and the control women. No consistency could be seen in diversity measurements. However, some tendencies could be seen regarding specific bacteria, as both genera *Collinsella* and *Blautia* were increased in the GDM group in three and four of the studies, respectively [54,58,60]. Additionally, genus *Sutterella* was reduced in the GDM women [54,60]. Three studies investigated changes in gut microbiota postpartum and found that a GDM specific gut microbiota could be identified within 18 months postpartum [60,61], but not five years postpartum [62]. None of the studies investigating the microbiota postpartum found significant differences in either beta diversity or alpha diversity.

Even though the studies did not agree on which specific bacteria that were significantly different in GDM, 14 of the studies observed associations between specific bacteria and different host parameters (Table 4) [40,45–48,50–54,56,57,59,60]. Most of the studies investigated the association between the gut microbiota and different glucose values, where eight studies found correlations between specific bacteria and fasting glucose levels [46,47,50–52,54,56,60], three studies found correlations with 1h OGTT values [50,52,56], and seven found correlations with 2h OGTT values [40,50,52,56,57,59,60] (Table 5).

**Table 4:** Major findings of the included studies.

Study	Increased in GDM	Decreased in GDM	Associations between bacteria and host parameters	Author conclusion
Mokkala et al.2020 [45]	**T1:**	**T1:**	*Holdemania filiformis*: ↑glucose *Alistipes shahii*: ↓glucose *Bifidobacterium bifidum*: ↓glucose	The specific gut microbiota species do not contribute to GDM in pregnant women with overweight or obesity. However, the gut microbiota of GDM women were less responsive to the diet intervention.
Ma et al.2020 [46]	**T1:** *Eisenbergiella*, *Lachnospiraceae NK4A136 group*, *Tyzzerella 4*	**T1:** *Dialister*, *eubacterium eligens group*, *eubacterium xylanophilum group*, *megasphaera*, *parabacteroides*, *parasutterella*, *Ruminococcaceae UCG 002*, *Ruminococcaceae UCG 003*, *Ruminococcaceae UCG 005*	*Eisenbergiella*: ↑FG *Tyzzerella*: ↑FG *Parabacteroides*: ↓FG *Parasutterella*: ↓FG *Ruminococcaceae UCG 002* ↓FG *Dialister*: ↓fasting insulin, ↓daily oil and yogurt intake.	The results demonstrated that aberrant gut microbiota interactions were associated with GDM before its onset, which was mainly reflected through the observed alterations in gut microbial composition and bacterial gene functions
Mokkala et al.2017 [47]	**T1:** *Clostridia*, *clostridiales*, *Ruminococcaceae*, *Ruminococcaceae;g_unclassified*, *Ruminococcaceae;g_unclassified;s_unclassified*	**T1:** *Bacteroidia*, *Bacteroidales*	*Rumninococcaceae*: ↑FG	The gut microbiota composition differs in women who developed GDM compared with women who did not develop GDM.
Gomez-Arango et al.2016 [48]	**T1:**	**T1:**	*Actinobacteria*: ↑insulin, ↑HOMA-IR *Tnericutes*: ↓insulin, ↓HOMA-IR *Coriobacteriaceae*: ↑insulin, ↑c-peptide *Collinsella*: ↑insulin, ↑HOMA-IR, ↑C-peptide, ↑maternal triglycerides, ↑VLDL cholesterol levels. *Ruminococcaceae*: ↑insulin, ↑leptin, ↓ fasting GIP, ↓resistin *Coprococcus*: ↑fasting GIP *Lachnospiraceae*: ↑leptin *Bacteroidaceae*: ↑ghrelin *Prevotellaceae*: ↓ghrelin	A relationship exists between the gut microbiome composition and the metabolic hormone milieu in early pregnancy.
Liu et al.2020 [40]	**T1:** **T2:** *Faecalibacterium*, *blautia*	**T1:** **T2:** *Bacteroidetes*, *Akkermansia*, *butyricimonas*, *ChristensenellaceaeR_7group*, *odoribacter*	*Faecalibacterium*: ↑2h OGTT, ↑IL-6, ↑TNF-α *Akkermansia*: ↓OGTT values *Butyricimonas*: ↓OGTT values *Christensenellaceae R-7 group*: ↓OGTT values *Odoribacter*: ↓OGTT values, ↓IL-8	There is an association between GDM and profound shifts in gut microbiota during T2. The specific bacterial patterns in the GDM women were correlated with blood glucose levels and inflammatory states.
Zheng et al.2020 [49]	**T1:** **T2:** *Eggerthella*, *Holdemania*, *megasphaera*	**T1:** *Coprococcus*, *desulfovibrio*, *Intestinimonas*, *Peptococcus*, *prevotella*, *Streptococcus*, *Bacilli*, *Lactobacillales*, *Streptococcaceae*, *veillonella* **T2:** *Coprococcus*, *Flavonifractor*, *Streptococcaceae*, *Streptococcus*	Did not detect any significant associations between microbial taxa and glucolipid measures, including fasting plasma glucose, lipid profiles, homeostatic model assessment-insulin resistance (HOMA-IR) score, and HOMA-cell index.	There are significant differences in the dynamics of gut microbiota from early to middle pregnancy between the groups. Women who develop GDM have reduced inter-time point variability in gut microbiota.
Koren et al.2012 [12]	**T1:** **T3:**	**T1:** **T3:**	NA	The study did not detect any differences between the microbiotas of GDM+ and GDM mothers.
Wang et al.2020 [50]	***T2*:** *Erysipelotrichaceae*, *prevotellaceae*, *Veillonellaceae*, *verrucomicrobiaceae*, *Akkermansia*, *allisonella*, *clostridium innocuum group*, *Lachnospiraceae UCG-010*, *prevotella 2*, *roseburia*, *terrisporobacter*, *Tyzzerella 3*, *unclassified_f_lachnospiraceae*, *lachnospiraceae*, *peptostreptococcaceae*	***T2*:** *Enterococcaceae*, *Ruminococcaceae*, *enterococcus*, *faecalibacterium*, *intestinibacter*, *klebsiella*, *Lachnospiraceae_N2004_group*, *norank_f_ruminococcaceae*, *Ruminococcus2*, *unclassified_f_enterobacteriaceae*, *lachnospiraceae*, *peptostreptococcaceae*	*Klebsiella*: ↑ FG at 12 wks. *Ruminococcus_2*: ↑ FG at 12 wks. *Norank_f__Ruminococcaceae*: ↓2h OGTT, ↑ FG at 12 wks. *Lachnospiraceae UCG-010*: ↑ 1h OGTT *Roseburia*: ↑ 1h OGTT *Prevotella 2*: ↑ 1h OGTT *Enterococcus*: ↓FG. ↓1h OGTT, ↓2h OGTT *Lachnospiraceae_NC2004_group*: ↓FG *Akkermansia*: ↓2h OGTT	GDM women have a significantly different microbial and metabolic signatures.
Chen et al.2020 [51]	**T2:** *Bacteroidetes*, *bacteroidia*, *Betaproteobacteria*, *atopobium*, *bacteroides*, *butyricimonas*, *Campylobacter*, *Dialister*, *f_Rikenellaceae;g_unclassified*, *odoribacter*, *Sutterella*, *unclassified_f_enterobacteriaceae*	**T2:** *Actinobacteria*, *Firmicutes*, *actinobacteria*, *clostridia*, *Erysipelotrichia*, *Bifidobacterium*, *f_coriobacteriaceae;g_unclassified*, *f_ruminococcaceae;g_unclassified*, *f_ruminococcaceae;g_unclassified*, *f_veillonellaceae;g_unclassified*, *gemmiger*, *o_clostridiales;f_unclassified;g_unclassified*, *oscillospira*, *unclassified_f_lachnospiraceae*	A module mostly of genera from *firmicutes*: ↓OGTT values A module of the genera *veillonella*, *haemophilus* and *rothia*: ↓FG Bacterial populations mostly of genera within the phylum *Firmicutes* (*Gemmiger*, *Oscillospira*, unassigned genera of *Clostridiales*, *Ruminococcaceae* and *Lachnospiraceae*): ↓ one or more OGTT values *Atopobium*: ↑glucose *Sutterella*: ↑glucose Unassigned genera of *Enterococcaceae*: ↑glucose	The study shows a relationship between changed gut microbiota composition in the second trimester of pregnancy before the diagnosis of GDM and fasting serum levels of metabolites.
*Kuang et al.2017 [52]	**T2:** *Megamonas*, *parabacteroides*, *phascolarctobacterium*, *Bacteroides sp*. *2_1_33B*, *Bacteroides sp*. *3_1_19*, *Bacteroides sp*. *D1*, *Bilophila sp*. *4_1_30*, *Capnocytophaga sp*. *oral taxon 338*, *Clostridium sp*. *L2-50*, *Coprobacillus sp*. *29_1*, *Coprococcus comes*, *Lachnospiraceae bacterium oral taxon 082*, *Lactobacillus fermentum*, *Lactobacillus salivarius*, *Megamonas funiformis*, *Megamonas rupellensis*, *Parabacteroides distasonis*, *Parabacteroides goldsteinii*, *Parabacteroides sp*. *2_1_7*, *Parabacteroides sp*. *20_3*, *Parabacteroides sp*. *D13*, *Parabacteroides sp*. *D25*, *Paraprevotella clara*, *Phascolarctobacterium succinatutens*, *Streptococcus agalactiae*, *Streptococcus anginosus*, *Weissella confusa*	**T2:** *Clostridiales*, *coriobacteriaceae*, *aggregatibacter*, *eggerthella*, *fusobacterium*, *haemophilus*, *mitsuokella*, *roseburia*, *ruminiclostridium*, *Aeromicrobium massiliense*, *Alistipes finegoldii*, *Alistipes senegalensis*, *Alistipes shahii*, *Bifidobacterium adolescentis*, *Bifidobacterium bifidum*, *Eubacterium siraeum*, *Intestinibacter bartlettii*, *Methanobrevibacter smithii*, *Roseburia intestinalis*, *Roseburia inulinivorans*	*Parabacteroides distasonis*: ↑2h OGTT *Klebsiella variicola*: ↑1h OGTT, ↑2h OGTT *Eubacterium rectale*: ↑1h OGTT *Lachnospiraceae bacterium 2-1-58FAA*: ↑FG, ↑1h OGTT *Catenibacterium mitsuokai*: ↑FG *Alistipes shahii*: ↓FG *Bacteroides*: ↓FG, ↓1h OGTT *Methanobrevibacter smithii*: ↓2h OGTT *Tannerella sp*. *6_1_58FAA_CT1*: ↓1h OGTT, ↓2h OGTT *Citrobacter freudii*: ↓1h OGTT *Eubacterium siraeum*: ↓FG *Eubacterium*: ↓FG, ↓1h OGTT, ↓2h OGTT *Alistipes senegalensis*: ↓FG, ↓1h OGTT, ↓2h OGTT *Eubacterium eligens*: ↓FG *Bacteroides sp*. *4_1_36*: ↓FG, ↓2h OGTT *Eggerthella spp*.: ↑ glucose tolerance *Megamonas spp*.: ↑ glucose tolerance *Allofustis seminis*: ↑ glucose tolerance Several species in *Lachnospiraceae*: ↑ glucose tolerance Several species in *Parabacteroides*: ↑ glucose tolerance Several *Alistipes* spp.: ↓glucose tolerance.	Women diagnosed with GDM suffered from moderate gut bacterial dysbiosis and functional dysbiosis that was not restricted to certain microbial species.
Li et al.2021 [54]	***T3*:** *Firmicutes*, *Clostridia*, *coriobacteriia*, *clostridiales*, *coriobacteriales*, *coriobacteriaceae*, *lachnospiraceae*, *Ruminococcaceae*, *blautia*, *collinsella*, *coprococcus*, *dorea*, *lachnospira*, *ruminococcus*, *blautia producta*, *clostridium spiroforme*, *collinsella aerofaciens*, *coprococcus catus*, *eubacterium dolichum*, *ruminococcus callidus*, *Ruminococcus gnavus*	***T3*:** *pyramidobacter piscolens*, *Bacteroidetes*, *bacteroidia*, *Betaproteobacteria*, *Bacteroidales*, *burkholderiales*, *alcaligenaceae*, *dethlosulfovibrionaceae*, *pyramidobacter*, *Sutterella*	*Clostridium spiroforme*: ↑FG *Eubacterium dolichum*: ↑FG *Ruminococcus gnavus*: ↑FG *Pyramidobacter piscolens*: ↓FG	This study showed a significantly difference in the gut microbiota between women with and without GDM in the third trimester of pregnancy.
Xu et al.2020 [53]	**T3:** *Gammaproteobacteria*, *haemophilus*, *Pasteurellales*, *Pasteurellaceae*	**T3:** *Rikenellaceae*, *Alistipese*, *phascolarctobacterium*	*Bifidobacterium*: ↓ maternal blood neutrophil counts, ↓ maternal white blood cell counts *Ruminococcus*: ↓ maternal blood neutrophil counts, ↓ maternal white blood cell counts *Gemmiger*: ↑ Neonatal body weight *Akkermansia*: ↓CRP	The maternal intestinal and oral microbiota at later pregnancy were significantly affected by GDM status.
Cui et al.2020 [55]	**T3:**	**T3:**	NA	The total faecal microbiota of healthy pregnant women and diseased pregnant women in the third trimester were similar, with no significant difference in gut microbiota.
Wu et al.2019 [56]	**T3:** *Bacteroides dorei*, *Bacteroides sp*. *3 1 33FAA*	**T3:** *Alistipes putredinis*, *Lactobacillus casei*	*Bacteroides dorei*: ↑FG, ↑1h OGTT *Alistipes putredinis*: ↓1h OGTT, ↓2h OGTT	GDM women showed greater between-individual diversity compared to the control group.
Liu et al.2019 [57]	**T3:** *Verrucomicrobiota*, *Akkermansia*	**T3:** *Faecalibacterium*	*Proteobacteria*: ↑TC, ↑LDL *Actinobacteria*: ↓2h OGTT *Faecalibacterium*: ↓TG, ↑LdMePE, ↑LPEt, ↑PG *Streptococcus*: ↑TC *Actinomyces*: ↑TC *Veillonella*: ↑TC, ↓HDL *Haemophilus*: ↑TC, ↓HDL *Firmicutes*: ↑3-Dehydrocarnitine, ↑CER, ↑DG, ↑Hexadecanamide, ↑Octadecanoicacid, ↑PEt, ↑PIP3 *Bacteroidetes*: ↓3-Dehydrocarnitine, ↓CER, ↓DG, ↓cPA, ↓Hexadecanamide, ↓PEt, ↓PIP3, ↓PC *Actinobacteria*:↓SO *Tenericutes*:↓SM *Prevotella*: ↑LPG, ↓PIP3	GDM women had a lower diversity of the gut microbiota.
Cortez et al.2018 [58]	**T3:** *Saccharibacteria*, *Gastranaerophilales*, *Christensenellaceae*, *Erysipelotrichaceae*, *lachnospiraceae*, *Peptococcaceae*, *Ruminococcaceae*, *Anaerostipes*, *bacteroides*, *Bifidobacterium*, *blautia*, *ChristensenellaceaeR_7group*, *Clostridiales-Family XIII AD3011 group*, *clostridium_sensu_stricto*, *collinsella*, *dorea*, *Eisenbergiella*, *Enterorhabdus*, *Lachnospiraceae NK4A136 group*, *Lachnospiraceae UCG-008*, *prevotella 9*, *roseburia*, *ruminiclostridium*, *ruminiclostridium 5*, *ruminiclostridium 9*, *Ruminococcaceae NK4A214 group*, *Ruminococcaceae UCG 002*, *Ruminococcaceae UCG 014*, *Ruminococcus1*, *Senegalimassilia*, *Subdoligranulum*, *Eubacterium coprostanoligenes*, *Eubacterium ruminantium*	**T3:** *Eubacterium rectale*	NA	The results show a tendency toward dysbiosis in the GDM condition, characterized by the presence of certain pathogenic genera and decreased diversity.
Wang et al.2018 [59]	**T3:** *Fusobacterium*	**T3:**	*Faecalibacterium/Fusobacterium*: ↓2h OGTT	The microbiota of pregnant women and neonates were altered in GDM, with a strong correlation between certain discriminatory bacteria and the oral glucose tolerance test.
*Crusell et al.2018 [60]	**T3:** *Granulicatella*, *Actinobacteria*, *actinobacteria*, *actinomycetales*, *coriobacteriales*, *coriobacteriaceae*, *leuconostocaceae*, *micrococcaceae*, *actinomyces*, *blautia*, *collinsella*, *desulfovibrio*, *leuconostoc*, *mogibacterium*, *Rothia*, *Ruminococcus2*, *faecalibacterium* **8 mo. pp.:** *Actinobacteria*, *actinobacteria*, *coriobacteriales*, *clostridiaceae_1*, *coriobacteriaceae*, *Alistipese*, *Anaerovovorax*, *clostridium_sensu_stricto*, *collinsella*, *dehalobacter*, *hafnia*, *Howardella*, *olsenella*, *Phreatobacter*, *faecalibacterium*	**T3:** *Marvinbryantia*, *Oscillibacter*, *faecalibacterium*, *acetivibrio*, *Anaerosporobacter*, *bacteroides*, *butyricicoccus*, *clostridium IV*, *Clostridium XVIII*, *Erysipelotrichaceae_incertae_sedis*, *Intestinimonas*, *Isobaculum*, *Sutterella*, *veillonella* **8 mo. pp.:** *Ruminococcus2*, *faecalibacterium*, *pseudomonadales*, *fusobacteriaceae*, *bacteroides*, *clostridium IV*, *eggerthella*, *fusobacterium*, *Isobaculum*, *Lachnospiracea_incertae_sedis*	*Collinsella*: ↑FG *Actinobacteria*: ↑FG *Butyricicoccus*: ↓insulin sensitivity *Prevotella*: ↑ 2h OGTT *Faecalitalea*: ↑2h OGTT *Verrucomicrobioales*: ↓insulin sensitivity *Verrucomicrobia*: ↓insulin sensitivity *Akkermansia*: ↓insulin sensitivity *Blautia* (OTU_2383, 3654, 140, 2684): ↑2h OGTT *Blautia* (OTU_2383, 3654, 140, 2684): ↑FG, ↓insulin sensitivity, ↓disposition index *Blautia* (OTU_486): ↓FG, ↓2h OGTT, ↑Insulin sensitivity *Escherichia/Shigella* (OTU_680, 361, 3): ↑disposition index, ↓insulin sensitivity, ↑FG, ↑2h OGTT *Clostridium IV* (OTU_68): ↓2h OGTT *Christensenella* (OTU_63): ↑FG, ↓weight gain *Bacteroides* (OTU_4999): ↑hsCRP *Alistipes* (OTU_98): ↓hsCRP *Anaerovorax* (OTU_538): ↓hsCRP *Acetivibrio*: ↓pre-pregnancy BMI *Leuconostoc*: ↓pre-pregnancy BMI *Clostridiales*(7 of 11 species): weight gain *Alistipes* (OTU_128): ↓weight gain *Eisenbergiella* (OTU_258): ↑Weight gain *Lactobacillus* (OTU_80): ↑weight gain	GDM diagnosed in late pregnancy is associated with an aberrant gut microbial composition at the time of diagnosis. About 8 months postpartum, the gut microbiota of previous GDM women is still different from women who had a normal pregnancy.
Fugmann et al. 2015 [61]	**8 mo. pp.:** *Rikenellaceae*, *Veillonellaceae*	**8 mo. pp.:** *Firmicutes*	NA	This study suggests that distinctive features of the intestinal microbiota are present in post-GDM women at risk for T2D.
Hasan et al.2018 [62]	**5 years pp:**	**5 years pp:**	**NA**	The study found no differences in the gut microbiota 5 years postpartum between women with and without GDM.
Hou et al. 2020 [63]	**?:** *Actinobacteria*, *Bacteroidetes*	**?:** *Firmicutes*	NA	GDM women had a different gut microbiota composition, but this was influenced by age.

Bacteria are illustrated if they are significantly different in relative abundance between cases and controls. ↑ indicates positively correlation between that bacteria and the host parameter, while ↓ indicates a negative correlation between the bacterium and the host parameter. Pp, postpartum; mo., months; T1, first trimester; T2, second trimester; T3, third trimester; FG, fasting glucose; HOMA-IR, Homeostatic Model Assessment for Insulin Resistance; VLDL, Very Low Density Lipoprotein; GIP, gastrointestinal polypeptide; IL-6, interleukin 6; OGTT, oral glucose tolerance test; TNF-α, tumor necrosis factor alpha; IL-8, interleukin 8; wks., weeks; CRP, C-reactive protein; TC, total cholesterol; LDL, low-density lipoprotein; LPEt, lysophosphatidylethanol; PG, phosphatidylglycerols; HDL, high-density lipoprotein; CER, ceramides; DG, diacylglycerols; PEt, phosphatidylethanol; PIP3, phosphatidylinositol 3; SO, sphingoshine; SM, sphingomyelins; LPG, lysophosphatidylglycerol; hsCRP, high-sensitivity C-reactive protein; cPA, cyclic phosphatidic acid; LdMePE, lysodimethylphosphatidylethanolamine.

**Table 5:** Correlations between specific bacteria and host glucose parameters.

Bacteria	Fasting glucose		1h OGTT		2h OGTT	
*Faecalibacterium/Fusobacterium ratio*						↓ [59]
*Module (veillonella*, *haemophilus* and *rothia)*		↓[51]				
*P_actinobacteria*	↑ [60]					↓ [57]
*P_actinobacteria; F_coriobacteriaceae; G_collinsella*	↑ [60]					
*P_bacteroidetes; F_bacteroidaceae; G_bacteroides*		↓ [52]		↓ [52]		
P_bacteroidetes; F_bacteroidaceae; G_bacteroides; S*_B*. *dorei*	↑ [56]		↑ [56]			
*P_bacteroidetes; F_bacteroidaceae; G_bacteroides; S_B*. *sp*. *4_1_36*		↓ [52]				↓ [52]
*P_bacteroidetes; F_Porphyromonadaceae; G_tannerella sp*. *6_1_58FAA_CT1*				↓ [52]		↓ [52]
*P_bacteroidetes; F_prevotellaceae; G_Prevotella*						↓ [60]
*P_bacteroidetes; F_prevotellaceae; G_Prevotella_2*	↑ [50]		↑ [50]			
*P_bacteroidetes; F_rikenellaceae; G_Alistipes; S_A*. *putredinis*				↓ [56]		↓ [56]
*P_bacteroidetes; F_rikenellaceae; G_Alistipes; S_A*. *senegalensis*		↓ [52]		↓ [52]		↓ [52]
*P_bacteroidetes; F_rikenellaceae; G_Alistipes; S_A*. *shahii*		↓ [52]				
*P_bacteroidetes; F_tannerellaceae; G_parabacteroides*		↓ [46]				
*P_bacteroidetes; F_tannerellaceae; G_parabacteroides; S_P*. *distasonis*					↑ [52]	
*P_euryarchaeota; F_methanobacteriaceae; G_methanobrevibacter; S_M*. *smithii*					↑ [52]	
*P_firmicutes; F_ Erysipelotrichaceae; G_ faecalitalea*					↑ [60]	
*P_firmicutes; F_ ruminococcaceae; G_Norank_f__Ruminococcaceae*	↑ [50]					↓ [50]
*P_firmicutes; F_christensenellaceae; G_christensenella*	↑ [60]					
*P_firmicutes; F_clostridiaceae; G_clostridium IV*						↓ [60]
*P_firmicutes; F_clostridiaceae; G_clostridium; S_C*.*spiroforme*	↑ [54]					
*P_firmicutes; F_enterococcaceae; G_Enterococcus*		↓ [50]		↓ [50]		↓ [50]
*P_firmicutes; F_Erysipelotrichidae; G_catenibacterium; S_C*.*mitsuokai*	↑ [52]					
*P_firmicutes; F_eubacteriaceae; G_eubacterium*		↓ [52]		↓ [52]		↓ [52]
*P_firmicutes; F_eubacteriaceae; G_eubacterium; S_E*. *dolichum*	↑ [54]					
*P_firmicutes; F_eubacteriaceae; G_eubacterium; S_E*. *eligens*		↓ [52]				
*P_firmicutes; F_eubacteriaceae; G_eubacterium; S_E*. *rectale*			↑ [52]			
*P_firmicutes; F_eubacteriaceae; G_eubacterium; S_E*. *siraeum*		↓ [52]				
*P_firmicutes; F_lachnospiraceae; G_blautia*	↑ [60]	↓ [60]			↑ [60]	↓ [60]
*P_firmicutes; F_lachnospiraceae; G_eisenbergiella*	↑ [46]					
*P_firmicutes; F_Lachnospiraceae; G_Lachnospiraceae UCG-010*			↑ [50]			
*P_firmicutes; F_Lachnospiraceae; G_Lachnospiraceae_NC2004_group*		↓ [50]				
*P_firmicutes; F_lachnospiraceae; G_roseburia*			↑ [50]			
*P_firmicutes; F_Lachnospiraceae; G_tyzzerella*	↑ [46]					
*P_firmicutes; F_Lachnospiraceae; S_Lachnospiraceae bacterium 2-1-58FAA*	↑ [52]		↑ [52]			
*P_firmicutes; F_peptostreptococcaceae; G_terrisporobacter*				↓ [50]		
*P_firmicutes; F_ruminococcaceae*	↑ [47] [50]					
*P_firmicutes; F_ruminococcaceae*, *G_ruminococaceae UCG 002*		↓ [46]				
*P_firmicutes; F_ruminococcaceae; G_faecalibacterium*					↑ [40]	
*P_firmicutes; F_ruminococcaceae; G_ruminococcus; S_R*. *gnavus*	↑ [54]					
*P_firmicutes; F_ruminococcaceae; G_ruminococcus2*	↑ [50]					
*P_proteobacteria; F_ Sutterellaceae; G_ parasutterella*		↓ [46]				
*P_proteobacteria; F_enterobacteriaceae G_citrobacter; S_C*. *freudii*				↓ [52]		
*P_proteobacteria; F_Enterobacteriasceae; G_Escherichia/Shigella*	↑ [60]				↑ [60]	
*P_Proteobacteria; F_enterococcaceae; G_Klebsiella*	↑ [50]					
*P_Proteobacteria; F_enterococcaceae; G_Klebsiella; S_K*. *variicola*			↑ [52]		↑ [52]	
*P_Synergistetes; F_Synergistaceae; G_pyramidobacter; S_P*. *piscolens*		↓ [54]				
*P_verrucomicrobia; F_verrucomicrobiaceae; G_Akkermansia*						↓ [50]

↑ indicates positively correlation between that bacteria and the host parameter, while ↓ indicates a negative correlation between the bacterium and the host parameter.

### Quality assessment of studies

Quality assessment of studies was performed using the NOS scoring system, and values are shown in Table 6. All studies, except two, received a NOS score above 5. Six received a score of 8 (high quality), as they included women both with and without risk factors for GDM development and matched cases and controls based on e.g. age, pre-pregnancy BMI, and gestational age, or made an adjustment for these confounding factors [40,46,49–51,53]. A moderate score of 6–7 was given to 13 studies, since these studies only included women in selected groups, such as moderate/high risk of developing GDM [45,47,48,57,60,61] or did not control for confounding factors [12,52,54,56,58,59]. The remaining two studies received a score of 4–5. Cui et *al*. [55] received a score of 5 as the study did not control for confounders, and their cases and controls were not well-defined [55]. Hou et *al*. [63] received a score of 4, as they did not control for confounders and did not describe collection time point, the DNA extraction method, or reference database used for taxonomic classification [63].

**Table 6:** Quality assessment of included studies based on the Newcastle–Ottawa Scale for case-control studies.

Study	Selection	Comparability	Exposure	Total
Ma et al. 2020 [46]	4	2	2	8
Liu et al. 2020 [40]	4	2	2	8
Zheng et al. 2020 [49]	4	2	2	8
Wang et al. 2020 [50]	4	2	2	8
Chen et al. 2020 [51]	4	2	2	8
Xu et al. 2020 [53]	4	2	2	8
Mokkala et al. 2020 [45]	3	2	2	7
Mokkala et al. 2017 [47]	3	2	2	7
Gomez-Arango et al. 2016 [48]	3	2	2	7
Crusell et al. 2018 [60]	3	2	2	7
Fugmann et al. 2015 [61]	3	2	2	7
Koren et al. 2012 [12]	4	0	2	6
Kuang et al. 2017 [52]	4	0	2	6
Li et al. 2021 [54]	4	0	2	6
Wu et al. 2019 [56]	4	0	2	6
Liu et al. 2019 [57]	3	1	2	6
Cortez et al. 2018 [58]	4	0	2	6
Wang et al. 2018 [59]	4	0	2	6
Hasan et al. 2018 [62]	3	1	2	6
Cui et al. 2020 [55]	3	0	2	5
Hou et al. 2020 [63]	3	0	1	4

## Discussion

Overall, most of the included studies found a significant difference in gut microbiota between GDM and non-GDM women. However, no clear conclusion could be drawn as no consistency could be seen in either diversity measurements or difference in the relative abundance of specific bacteria across the studies. Furthermore, it is still uncertain when the dysbiosis develops, and whether the altered microbiota is part of the cause or the consequence of the GDM development. Only a few of the included studies found a GDM-associated gut microbiota early in the pregnancy, suggesting that dysbiosis may be developed later in the pregnancy. Instead most studies agree that a state of dysbiosis is present in the second and third trimester of pregnancy, though it is most pronounced in the second trimester.

There are some important factors in study design that might influence the results. Major confounders include differences in GDM manifestation and treatment between the trimesters. In the third trimester of the pregnancy, it is expected that all the women with GDM receive treatment in some form, whether it being based on diet changes, exercise, medication or a combination of these. All these factors alone could affect the gut [64–66]. There are, however, great differences in which treatment regimens the included women have received. Some of the reviewed studies include women receiving both lifestyle counseling and antidiabetic drugs [53,56,58], while others exclude women taking antidiabetic treatment such as insulin and metformin [45,48,52,60,62,63]. A recent study showed that GDM patients adherent to the diet recommendations had a lower relative abundance of Bacteroides [64]. Moreover, the gut microbiota in GDM women with successful glycemic control after lifestyle intervention differs from that in GDM women with failure in glycemic control [65]. This indicates that treatment with lifestyle intervention, and how the women respond to the treatment, have a confounding effect in the studies investigating the microbiota after GDM diagnosis. Changes in the gut microbiota towards that found in healthy controls have also been described in T2DM patients’ following antidiabetic treatment [66]. This could also be the case in GDM studies that include women using antidiabetic drugs. Therefore, differences in GDM treatment could explain the highly heterogenous results reported from third trimester studies. In the second trimester, on the other hand, fecal samples were collected after/around GDM diagnosis, but before initiation of GDM treatment. These therefore represent a more homogeneous study population, which also appears to be reflected in the higher agreement between studies. However, this must be interpreted with caution, as all the second trimester studies are performed in China, thereby being more similar regarding ethnical factors. Importantly, it has previously been shown that the country of origin had a major influence on the gut microbiota composition, while the diabetic status of the participant only had a minor effect [67].

When comparing changes across the different trimesters, it is important to consider the hormonal changes that happens throughout the pregnancy. During a normal pregnancy both progesterone and estrogen levels increase dramatically, especially, in the second trimester. These hormones have both been linked to bacterial changes in the gut [3,68] and could therefore contribute to the heterogenous results seen across the different trimesters. Additionally, these hormones have also been found to be increased in GDM women, though estrogen were only increased compared to a subgroup of the healthy control women (low risk of developing GDM) [69]. Therefore, estrogen and progesterone might contribute to the gut microbiota dysbiosis in the second trimester. However, none of the included studies have compared these hormones between GDM and non-GDM women and the effect of these hormones on the gut microbiota is therefore uncertain.

An important factor in the definition of cases and controls is the used diagnostic criteria for GDM. Most of the studies use the diagnosis criteria from IADPSG, but the Finnish studies used higher glycemic cutoffs to diagnose GDM. Using different tests and different criteria will influence which women are diagnosed with GDM. Thus, a woman could be diagnosed with GDM in one study and grouped as a healthy control in another study, indicating a need for a general approach to diagnosing GDM. However, this might not be possible, as the IADPSG criteria for example seem to be unsuitable to use on Danish women, as these women have higher fasting blood glucose without being at increased risk of pregnancy complications [70]. This is further supported by a recent Cochran review that concluded that there was insufficient evidence to suggest which strategy is best for diagnosing GDM [71]. Only two studies, besides studies from China, reported fasting blood glucose values for their healthy controls, which makes it difficult to compare fasting blood glucose in healthy controls between the different ethnicities. However, it seems that the controls from the Chinese studies did have a lower fasting blood glucose compared with the two studies from North Europe. The reason for this is not fully understood, but might be explained by differences in lifestyle, diet, and genetics.

The inconsistency between the studies could also be due to the selection criteria for the cases and controls. Obesity is a known risk factor for development of GDM [37] and therefore an overrepresentation of obese participants might be present in the GDM group. Difference in BMI between the cases and controls could pose a problem, as pre-pregnancy BMI has shown to have a confounding effect on the gut microbiota [60,72]. Overall, the GDM women from the included studies had a higher pre-pregnancy BMI compared to the control group and only a few of the studies either matched their participants [40,51] or made adjustment [45,47,60] for pre-pregnancy BMI. Some of the studies that did not match their groups or made adjustments also found that the pre-pregnancy BMI was significantly higher in the GDM group [52,58,60,63]. Therefore, a confounding effect of BMI might be present in some of the studies, which could contribute to the heterogenous results between the studies. Furthermore, we observed that the BMI levels were much lower in the Chinese studies compared to the studies from Finland or Denmark, which again made direct comparison difficult.

The use of antibiotics is likely to impact the gut microbiota and thereby contribute to the inconsistency between the studies. Treatment with antibiotics have shown to have long-lasting effects on the gut microbiota, even though most of the microbiota composition can be restored after 1.5 months [73]. The duration of the washout period before sample collection varied among the studies, and a confounding effect of antibiotics might influence the gut microbiota in the studies with short washout period [12,47,50,52–55,61,63]. For instance, treatment with antibiotics was not an exclusion criterion during the first and the second trimester in the study by Koren *et al*. [12]. Therefore, the possible differences between GDM and non-GDM women present in the first trimester might be diminished.

Finally, the inconsistency among the studies could also be due to the various procedures for sample handling and methodology, since sample storage, DNA extraction procedure, 16S rRNA target region, and reference database all have been shown to have a pronounced effect on the gut microbiota. None of the included studies have used the same approach, which may explain some of the heterogeneous results. For instance, various storage procedures were used, where most studies froze the samples after collection, but a few studies used storage buffers [49,61,62]. In particular, the use of storage buffer has been shown to induce changes in the gut microbiota [74]. Furthermore, DNA extraction procedures have also been observed to affect bacterial composition. For instance, some bacteria are difficult to lyse and might be underestimated in some analyses due to the extraction method [75,76]. Since the included studies use very different protocols for DNA extraction, this could make it difficult to compare the studies. Moreover, a previous study has also shown that the choice of target region on the 16S gene could affect the detection of specific bacteria [77]. Furthermore, the methods used for taxonomic assignment of the sequence reads could also influence the results. The metagenomic studies have a high resolution, where it is possible to identify bacteria at species level. Three of the studies using 16S rRNA sequencing also identified taxa at species level [54,57,61]. Therefore, a comparison at species level was not possible for all the included studies. Even though all the studies using 16S rRNA sequencing clustered their sequencing reads into OTUs, it can still be difficult to compare between studies due to differences in sequence similarity thresholds. For instance, Ma *et al*. 2020 [46] used a similarity threshold of 100% meaning that they only clustered reads that were 100% identical. Therefore, it would be expected that Ma *et al*. 2020 [46] had a higher number of different OTUs compared to the studies that cluster their reads into 97% OTUs. In addition, the choice of reference database could also influence the results. For instance, the Greengenes database had difficulties in predicting the actual number of genera, while the Silva database was prone to false-positive results [78]. This could be a problem in this review since SILVA [40,45,46,57,58,62] and Greengenes [12,48,51,54,55,59] were the two most applied reference databases among the included studies.

Additionally, the use of different alpha and beta diversity measurements could also influence the results. For instance, Xu *et al*. 2020 [53] and Ma *et al*. 2020 [46] applied Bray Curtis, weighted and unweighted UniFrac to investigate beta diversity. Both studies found a significant difference in beta diversity when using unweighted UniFrac, but not when using the two other methods. Unweighted UniFrac is sensitive to the absence and presence of low abundant bacteria, while both weighted UniFrac and Bray Curtis are more sensitive to the more abundant bacteria. Therefore, it could indicate that the gut microbiota between GDM and control women in the studies by Xu *et al*. 2020 [53] and Ma *et al*. 2020 [46] are very similar in bacteria composition when looking at the most abundant bacteria, but differ when looking at the presence/absence of low abundance bacteria. However, some of the included studies that did not report significant difference in beta diversity have only used the Bray-Curtus method [40,45,52,55,56,61,62] or the Weighted UniFrac [49,51,60]. It is therefore possible that a significant difference could be seen in these studies if they included other measures for beta diversity. The same could apply for alpha diversity. Shannon diversity and Chao1 richness were the most applied methods to investigate alpha diversity, but they were not always used together [45,49,51,52,56,57,60,62] Shannon diversity takes both species richness and evenness into account, while Chao1 richness only accounts for richness. If two samples have the same richness but different relative abundance of the bacteria, it is possible to see a difference with Shannon diversity, but not with Chao1 richness. For instance, Li *et al*. 2021 [54] showed a significant difference in Shannon diversity, but no difference in Chao1 richness, which is the opposite in Liu *et al*. 2019 [57]. More of the included studies only used Shannon diversity to investigate alpha diversity and it is therefore unknown if a significant difference could be found if they had used other alpha diversity analyses. Notably, different cut-off thresholds for significance were used by the individual studies, again making direct comparisons difficult.

Even though the studies showed very inconsistent results, *Collinsella* and *Blautia* showed a tendency to be increased in GDM women. These genera are both positively correlated with T2DM [7,79,80] and could therefore be involved in the development or maintenance of a diabetic state. This is further supported by the metabolic changes associated with these two bacteria. For instance, *Collinsella* has been positively associated with insulin level and Homeostatic Model Assessment for Insulin Resistance (HOMA-IR) and negatively associated with insulin sensitivity [81,82]. Therefore, a high relative abundance of *Collinsella* might contribute to insulin resistance and the development of GDM. *Collinsella* has, furthermore, been reported to be sensitive to diet and weight loss [81,82], which is the first line of GDM treatment. This might explain why only a few studies see an increase in the relative abundance of *Collinsella* in the third trimester of pregnancy. The same could apply for *Blautia*, as *Blautia* is also influenced by dietary intake and has also been positively correlated to fasting plasma glucose and insulin levels [80,82,83]. Therefore, both *Colinsella* and *Blautia* might be a part of the GDM-associated gut microbiota, where they contribute to maintaining a diabetic state.

Most of the included studies found a correlation between specific bacteria and host glucose metabolism. Interestingly, eight of the genera that correlated to fasting glucose, 1h OGTT or 2 h OGTT levels coincided with the bacteria that were found to be statistically significantly different in relative abundance between cases and controls in three or more of the included studies (Table 3). This could support the role of these bacteria in the glucose metabolism. However, the influence *Blautia* could have on the development/maintenance of GDM might be species specific as the study by Crusell *et al*. 2020 [60] found that some *Blautia* OTUs were positively correlated with increased blood glucose and reduced insulin sensitivity, while other *Blautia* OTUs had the opposite correlation. This is further supported by the correlations related to the genus *Eubacterium* and species belonging to this bacterium. For instance, *Eubacterium dolichum* was positively correlated with fasting glucose, while *Eubacterium eligens* was negatively correlated with fasting glucose. If the function of the bacteria is mostly decided at species level, this could also explain the inconsistent results, when comparing GDM and non-GDM women at genus level in this review. Limitations of the current review should be considered. First, the studies are very heterogeneous regarding methodology and demography, which makes it difficult to compare results between the studies and likely contributes to some of the differences in the results. This also made it difficult to combine the results into a meta-analysis. Secondly, the risk of bias in many of the studies was high, as less than half of the studies either matched their participants or adjusted for confounding factors. Publication bias should also be taken into consideration, even though some of the included studies did report negative findings. Furthermore, language bias cannot be excluded, as we only included articles written in English and Danish.

## Conclusion

This systematic review has shown that although most of the studies found an association between GDM and gut microbiota dysbiosis, no GDM-specific gut microbiota could be identified. All studies in the second trimester found a difference between GDM and non-GDM women, indicating that dysbiosis is present at the time of diagnosis. Nevertheless, it is still unclear when the dysbiosis develops, as no consensus could be seen between the studies investigating the gut microbiota in the first trimester of pregnancy. However, studies varied widely concerning methodology and study design, which might explain the highly heterogeneous gut microbiota compositions between studies. Furthermore, only three studies investigated the gut microbiota postpartum, which made it difficult to determine if the GDM women have gut microbiota dysbiosis after birth. Therefore, future studies seeking to determine the role of gut microbiota in GDM and the increased risk of T2DM, need to investigate multiple time points (before, during, and after pregnancy) and consider possible confounding factors such as age, ethnicity, pre-pregnancy BMI, and treatment (lifestyle and/or medicine).

## Supporting information

S1 Table Gut microbiota data for studies included in the systematic review. (XLSX)

S2 Table Quality assessment of studies included in the systematic review. (XLSX)

S1 File The search strategies for Embase and PubMed. (PDF)

S2 File PRISMA checklist. (PDF)
