# Supplementary material for: Characteristics of the gut microbiome in women with gestational diabetes mellitus: A systematic review
Source: PLoS One. 2022 Jan 13;17(1):e0262618. doi: 10.1371/journal.pone.0262618 (PMC8757951; doi:10.1371/journal.pone.0262618)
Supplement: S1 File — (PDF) [file pone.0262618.s003.pdf]

S1 File

The search strategies for Embase and PubMed are described below:

Database: Embase <1974 to 2021 February 24> Search Strategy:

1 exp microflora/ (133318)
2 exp bacterium/ (1633776)
3 dysbiosis/ (7490)
4 (microflora* or microorganism* or "16s rRNA" or microbial* or eubios* or dysbios* or microbiome* or microbiota* or
bacteria* or Dysbacterios* or disbios* or disbacterios*).ti,ab,kw. (1105508)
5 1 or 2 or 3 or 4 (2140383)
6 exp pregnancy diabetes mellitus/ (37686)
7 (Gestational Diabet* or pregnancy induced diabet* or gestational hyperglycaemia* or gestational hyperglycemia* or
gdm).ti,ab,kw. (27578)
8 exp pregnancy/ (686724)
9 (gestational or pregnan*).ti,ab,kw. (749137)
10 8 or 9 (1002053)
11 hyperglycemia/ (100364)
12 (hyperglycaemia* or hyperglycemia*).ti,ab,kw. (84327)
13 glucose intolerance/ (19159)
14 exp glucose tolerance test/ (61123)
15 ogtt.ti,ab,kw. (17176)
16 glucose blood level/ (262070)
17 glucose level*.ti,ab,kw. (75056)
18 Glucose Intolerance.ti,ab,kw. (15816)
19 prediabetic*.ti,ab,kw. (4645)
20 or/11-19 (414503)
21 10 and 20 (25466)
22 6 or 7 or 21 (53034)
23 5 and 22 (987)
24 conference*.pt. (4813680)
25 23 not 24 (785)

Database: PubMed <1000 to 2021 February 24> Search Strategy

((((((((("Pregnancy"[Mesh] OR gestational[Text Word] OR pregnan*[Text Word])) AND (((((((prediabetic*[tw]) OR glycemic level*[tw]) OR (Glucose Tolerance Test*[tw] OR ogtt[tw])) OR "Glucose Tolerance Test"[Mesh]) OR Glucose Intolerance*[tw]) OR Glucose Intolerance*[tw]) OR "Glucose Intolerance"[Mesh]) OR ("Hyperglycemia"[Mesh] OR hyperglycaemia*[tw] OR hyperglycemia*[tw])))) OR "Diabetes, Gestational"[Mesh]) OR Gestational Diabet*[Text Word]) OR pregnancy induced diabet*[Text Word]) OR gestational hyperglycaemia*[tw]) OR gestational hyperglycemia*[tw])) AND (((((((((((microorganism*[tw]) OR "16s rRNA"[tw]) OR microbial*[Text Word]) OR eubios*[tw]) OR ((dysbios*[tw] OR Dysbacterios*[tw] OR disbios*[tw] OR disbacterios*[tw]))) OR "Dysbiosis"[Mesh]) OR Microflora*[Text Word]) OR (microbiome*[Text Word] OR microbiota*[Text Word])) OR "Microbiota"[Mesh]) OR "Bacteria"[Mesh])) OR bacteria*[Text Word])
